# Supplementary material for: Iterative improvement in the automatic modular design of robot swarms
Source: PeerJ Comput Sci. 2020 Dec 7;6:e322. doi: 10.7717/peerj-cs.322 (PMC7924708; doi:10.7717/peerj-cs.322)
Supplement: Supplemental Information 3 [file peerj-cs-06-322-s003.zip › argos3/doc/api/standalone/a00493_source.html]

ARGoS: plugins/robots/generic/control\_interface/ci\_differential\_steering\_actuator.h Source File


- Main Page
- Related Pages
- Namespaces
- Classes
- Files

- File List
- File Members

# plugins/robots/generic/control\_interface/ci\_differential\_steering\_actuator.h

Go to the documentation of this file.

```
00001 
00007 #ifndef CCI_DIFFERENTIAL_STEERING_ACTUATOR_H
00008 #define CCI_DIFFERENTIAL_STEERING_ACTUATOR_H
00009 
00010 /* To avoid dependency problems when including */
00011 namespace argos {
00012    class CCI_DifferentialSteeringActuator;
00013 }
00014 
00015 #include <argos3/core/control_interface/ci_actuator.h>
00016 
00017 namespace argos {
00018 
00019    class CCI_DifferentialSteeringActuator : public CCI_Actuator {
00020 
00021    public:
00022 
00023       virtual ~CCI_DifferentialSteeringActuator() {}
00024 
00025       virtual void SetLinearVelocity(Real f_left_velocity,
00026                                      Real f_right_velocity) = 0;
00027 
00028 #ifdef ARGOS_WITH_LUA
00029       virtual void CreateLuaState(lua_State* pt_lua_state);
00030 #endif
00031 
00032    protected:
00033 
00034       Real m_fCurrentVelocity[2];
00035 
00036    };
00037 
00038 }
00039 
00040 #endif
```

---

Generated on 10 Jul 2018 for ARGoS by 
 1.6.1
